# A specific protein-enriched enteral formula decreases cortisolemia and improves plasma albumin and amino acid concentrations in elderly patients

**DOI:** 10.1186/1743-7075-7-58

**Published:** 2010-07-13

**Authors:** Josune Olza, María D Mesa, Rafael M Poyatos, Concepción M Aguilera, Rosario Moreno-Torres, Milagros Pérez, Antonio Pérez de la Cruz, Angel Gil

**Affiliations:** 1 Department of Biochemistry and Molecular Biology II, Institute of Nutrition and Food Technology, Biomedical Research Center, University of Granada, Granada, Spain; 2 Clinical Analysis Service, University Hospital Virgen de las Nieves, Granada, Spain; 3 Clinical Nutrition and Dietetic Unit, University Hospital Virgen de las Nieves, Granada, Spain; 4 Department of I+D Vegenat S.A, Pueblonuevo del Guadiana, Badajoz, Spain

## Abstract

**Background:**

Old age is associated with an involuntary and progressive but physiological loss of muscle mass. The aim of this study was to evaluate the effects of exclusive consumption for 6 months of a protein-enriched enteral diet with a relatively high content of branched-chain amino acids on albuminemia, cortisolemia, plasma amino acids, insulin resistance, and inflammation biomarkers in elderly patients.

**Methods:**

Thirty-two patients from the Clinical Nutrition Outpatient Unit at our hospital exclusively consumed a protein-enriched enteral diet for 6 months. Data were collected at baseline and at 3 and 6 months on anthropometric and biochemical parameters and on plasma concentrations of amino acids, cortisol, adrenocorticotropic hormone, urea, creatinine, insulin resistance, and inflammation biomarkers.

**Results:**

The percentage of patients with albumin concentration below normal cut-off values decreased from 18% to 0% by the end of the study. At 6 months, concentrations of total plasma (*p *= 0.008) and essential amino acids (*p *= 0.011), especially branched-chain amino acids (*p *= 0.031), were higher versus baseline values, whereas 3-methylhistidine (*p *= 0.001), cortisol (*p *= 0.001) and adrenocorticotropic hormone (*p *= 0.004) levels were lower.

**Conclusions:**

Regular intake of specific protein-enriched enteral formula increases plasma essential amino acids, especially branched-chain amino acids, and decreases cortisol and 3-methylhistidine, while plasma urea and creatinine remain unchanged.

## Background

The proportion of the elderly is high and steadily rising in Western populations [1]. Aging is associated with an involuntary and progressive but physiological loss of muscle mass, designated sarcopenia [2], which is currently considered an emerging problem of Public Health [1]. Sarcopenia is accompanied by a reduction in strength and quality that leads to muscle weakness, limiting mobility and increasing vulnerability to injury [1,3]. Reduced muscle mass in older adults has also been associated with susceptibility to disease and decreased survival rates after critical illness [2].

Skeletal muscle is the body's main reservoir of amino acids, which contain 50-75% of human body proteins. Skeletal muscle is a vital supplier of amino acids for use as fuel by the brain and immune system and as a substrate for tissue repair during malnutrition, injury, and disease [4]. It is important to maintain the body protein mass in order to live well and remain physically independent.

It has been postulated that age-related muscle mass reduction is due to a multi-factorial process that include genetic and other factors such as cellular apoptosis, changes in protein metabolism, hormonal alterations, loss of neuromuscular function, inappropriate nutrition, lack of regular physical activity, and diseases or their sequelae [5]. The mechanisms underlying the loss of skeletal muscle are not clear but can be linked to a preceding disruption in the regulation of muscle protein turnover, specifically to an imbalance between protein synthesis and breakdown [6]. It has been demonstrated that the combination of prolonged inactivity and hypercortisolemia increases muscle protein catabolism and reduces muscle protein synthesis, even when substrates are available [6]. Likewise, hypercortisolemia has been observed in hypoalbuminemic individuals [7]. Albumin is a good marker of protein malnutrition, and lower concentrations are commonly observed in older subjects and have been associated with poor health outcomes and mortality [8].

Inflammatory cytokines such as interleukin (IL)-6 and tumor necrosis factor alpha (TNF-α) contribute, together with a reduced concentration of growth factors, to the development of sarcopenia [1]. Insulin resistance (IR) is also implicated in sarcopenia and is frequently observed in elderly subjects, although it is mostly associated with obesity and, recently, with sarcopenic obesity [9].

Co-ingestion of protein and leucine stimulates muscle protein synthesis rates to the same extent in young and elderly lean men [10], and the intake of nutrients and proteins affects the albumin synthesis rate in humans [11]. Ingestion of 15 g/d of whey protein, containing 6.5 g of essential amino acids (EAA), has been reported to be strongly anabolic to skeletal muscle in healthy older individuals [12]. Enteral nutrition (EN) formulas are prescribed to elderly patients, when it is necessary, as an exclusive diet or in combination with other foods to achieve recommended dietary intakes. With this background, the aim of this study was to evaluate the effect of a six-month exclusive protein-enriched enteral diet with a relatively high content of branched-chain amino acids (BCAA) and other essential amino acids on albuminemia, cortisolemia, plasma amino acids, IR, and inflammation biomarkers in elderly patients subjected to total enteral nutrition.

## Methods

### Study design

An experimental, prospective, intention-to-treat clinical trial was performed in elderly patients recruited from the Clinical Nutrition and Dietetics Outpatient Unit of the Virgen de las Nieves University Hospital (Granada, Spain). The patients were fed exclusively on an essential amino acid-enriched enteral diet for 6 months. The protein blend comprised 50% caseinate, 25% whey protein, and 25% pea protein (T-Diet Plus^®^, from Vegenat S.A.). A previous study reported the protein efficiency ratio (PER) of this blend to be 4.04 and the apparent digestive coefficient to be 93.91% [13].

Patients received 1500 mL of this diet to meet their daily energy and nutritional requirements. The actual mean daily intake for the whole study period was 1266 ± 59 kcal. Table 1 shows the nutritional composition of the enriched protein enteral diet and Table 2 the amino acid composition. The diet was administered as a bolus using a nasogastric tube or *via *a stoma with a large-bore syringe. All patients received 1000-1200 mL of water daily to maintain optimal hydration status.

**Table 1:** Nutritional composition of the specific protein-enriched enteral formula*

Nutrients		Per 100 mL
Energy	kcal	100
Protein	g	4.00
Casein	g	2.00
Whey proteins	g	1.00
Pea proteins	g	1.00
Total Carbohydrates	g	12.3
Total Fat	g	3.90
Fiber	g	1.70
Total Minerals	g	0.57

*The product as it is marketed (T-Diet Plus^®^) contains a vitamin complex to satisfy 100% of vitamin reference intake for elderly, assumed a daily intake of 1500 kcal (6276 kJ).

**Table 2:** Amino acid composition of the specific protein-enriched enteral formula

Amino acid	g/100 g protein
Alanine	4.02
Arginine	4.92
Aspartic Acid*	9.20
Cystine	1.05
Glutamic Acid*	22.68
Glycine	2.50
Histidine	2.52
Isoleucine	5.52
Leucine	9.67
Lysine	8.32
Methionine	2.47
Phenylalanine	4.87
Proline	9.09
Serine	5.27
Threonine	4.85
Tryptophan	1.32
Tyrosine	4.57
Valine	6.10
Branched-chain amino acids	21.29
Methionine + Cysteine	3.52
Phenylalanine + Tyrosine	9.44

* These values include asparragine and glutamine, which are converted to aspartic and glutamic acid, respectively, during the protein hydrolysis carried out in the analytical procedure

### Subjects

Thirty-two patients (25 female, 7 male; mean age 70.1 ± 3.7 yrs) from the Clinical Nutrition and Dietetics Outpatient Unit of our hospital participated in the study. Inclusion criteria were: age >65 years, prescription of total enteral nutrition for ≥6 months, and voluntary consent to participate. Exclusion criteria were: unstable clinical condition, fatal illness, inclusion in other clinical trial, or refusal to participate. The patient or next of kin was informed about the purpose and procedures of the study before written consent was obtained. The protocol was performed in accordance with the *Declaration of Helsinki *and approved by the Ethics Committee of our hospital. At the end of the experimental period, only 17 of the 32 enrolled patients remained in the study; 15 patients were lost to the study due to: refusal to continue in the study (n = 6), death (n = 4), change in diet (n = 3) (two moved to a nursing home in other province, and one was prescribed a specific formula for diabetics due to his clinical condition), and withdrawal of enteral nutrition (n = 2). Table 3 shows the baseline demographic and anthropometric characteristics of the subjects and their concomitant diseases and medication. A large proportion of patients had cognitive deficits and a history of cerebrovascular diseases and cardiovascular events. The majority were receiving gastric protectors and a large proportion psycho-drugs and anticoagulants.

**Table 3:** Baseline demographic and anthropometric characteristics of the subjects, concomitant diseases and medication

Demographic and anthropometric characteristics		
Age (y)		70.1 ± 3.7 *
Sex	Female	25
	Male	7
Tricipital Skinfold (mm)		17.6 ± 2.2
Mid Arm circumference (cm)		24.3 ± 1.0

Concomitant diseases (%)		

Cognitive deficits and Alzheimer disease		52.9
Cerebrovascular diseases and cardiovascular events		41.2
Other causes (cancer, accidents)		17.6

Medication (%)		

Gastric protectors		82.3
Psychodrugs		40.0
Anticoagulants		41.2
Antihypertension medication		25.0
Diuretics		30.0
Analgesics		20.0
Antiarrhythmics		15.0
Antidiabetics		5.0

*x ± SEM (for all such values).

### Anthropometric measurements

No attempt was made to measure the weight or height of the patients, who were mostly bed-bound. Measurements were taken of the mid-arm circumference (MAC) and tricipital skinfold (TS). The MAC (in cm) was measured midway between the tips of the acromion and olecranon processes, using a flexible tape measure. The TS (in mm) was taken on the dorsal arm midway between the tips of the acromion and olecranon, using a caliper. These measurements were compared with reference tables for the elderly Spanish population classified by age and sex [14].

### Blood samples

Fasting blood samples were obtained from patients between 8:00 and 10:00 am after 8-10 h overnight fasting at baseline (before diet) and again after 3 and 6 months of the study diet. Serum and plasma (EDTA-coated tubes from BD Vacutainer, Plymouth, UK) were separated by centrifugation (15 min at 1750 g) and immediately processed or divided into aliquots and frozen at -80°C until their analysis.

### Biochemical analysis

Plasma urea (Coefficient of Variation [CV 2.8%]), creatinine (CV 3.2%), albumin (CV 3%), and glucose (CV 1%) were determined by standardized spectrophotometric techniques using a Roche Hitachi Modular DDP clinical analyzer system (Roche Diagnostics Spain, S.L., Barcelona). Cortisol (CV 1.7%) and adrenocorticotropin hormone (ACTH) (CV 8.9%) were determined by chemiluminescence using a LIAISON Immunoassay Analyzer (Soaring S.T.A, Saluggia, Italy). Fasting insulin (CV 2.6%) was analyzed by standardized electrochemiluminescence using an E-170 Elecsys Modular Analytics system (Roche Diagnostics España, S.L., Barcelona). All biochemical analyses were performed in the Virgen de las Nieves University Hospital. IR was calculated by using the homeostatic assessment model (HOMA-IR), defined by the equation HOMA-IR = fasting glucose (mM) × fasting insulin (μU/mL)/22.5.

### Inflammatory biomarkers

LINCOplex™kits (Linco Research, MO) were used on a Luminex^® ^200™System (Luminex Corporation, TX) to determine IL-6 (CV 7.8%), IL-8 (CV 7.9%), TNF-α (CV 7.8%) (Cat. #HADK2-61K-B), and adiponectin (CV 9.2%) (Cat. #HCVD1-67AK). C-reactive protein (C-RP) (CV 4%) was determined with a particle-enhanced turbidimetric immunoassay (PETIA) (Dade Behring Inc., IL).

### Amino acid analysis

Plasma amino acids were determined by high-performance liquid chromatography (HPLC) using a Beckman system gold HPLC (Beckman Coulter Inc. Fullerton, CA), which includes a solvent module 126AA, post column reactor 232, detector 168, autosampler 508, and a 3×250-mm spherogel AA lithium column (sulfonated polystyrene-divinylbenzene copolymers), as previously described by Le Boucher *et al *[15]. Lithium column eluents Li-292, Li-365, and Li-375 and trione ninhydrin reagent were from Pickering Laboratories (Mountain View, CA), and norleucine was from Sigma (St. Louis, MO).

### Statistical analysis

Plasma cortisol, ACTH, urea, creatinine, IR, inflammation biomarkers, and amino acids are expressed as mean values with standard error of the mean (SEM). The anthropometric and albumin values are expressed as a percentage of reference values for healthy individuals [14]. Variables were checked for normality and homogeneous variance by using the Kolmogorov-Smirnov and the Levene tests, respectively. As most of the data did not follow a normal distribution, the non-parametric Friedman test for three repeated measures was used to assess differences as a function of treatment time. The Wilcoxon test was used for *post hoc *comparisons to analyze specific differences between times. Percentages were compared using a chi-square test. Correlations between different variables were assessed using the rank Spearman test. A value of *p < 0.05 *was considered significant. SPSS 15.01 for Windows (SPSS Inc., Chicago, IL) was used for the data analyses.

## Results

No significant differences in anthropometric measurements were found between before and after consumption of the enriched-protein enteral formula for 6 months. However, the percentage of subjects with albumin below the normal plasma cut-off value fell from 18% at baseline to 0% at 6 months (Table 4).

**Table 4:** Albumin and anthropometric measurements below the normal range in elderly patients receiving a protein-enriched enteral diet for 6 months

Percentage of patients	0 mo	3 mo	6 mo	P*
Albumin	18.8	6.3	0	0.060
Mid-arm circumference	31.3	23.5	29.4	0.874
Triceps skinfold	37.5	41.2	41.2	0.918

Undernutrition was defined by plasma albumin < 3 g/dL, mid-arm circumference < 25th percentile, and triceps skinfold < 25th percentile by age and sex in the elderly [14].

*Differences among sample times were tested using the chi-square test, considering P < 0.05 to be significant.

**Figure 1:**
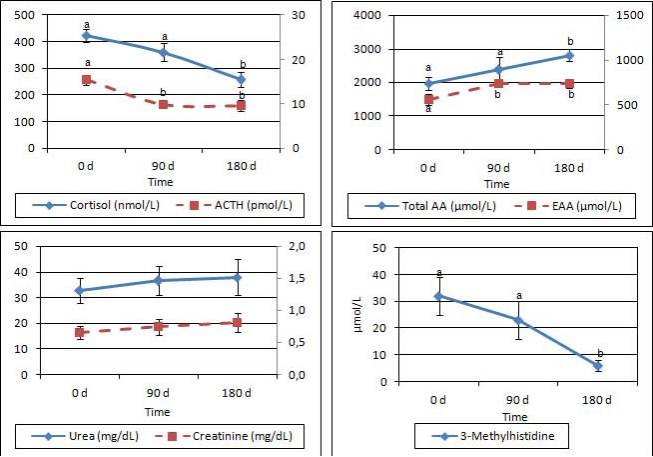
**Plasma concentrations of cortisol and acetylcholine (A); total and essential amino acids (B); urea and creatinine (C); and 3-methylhistidine (D) at baseline and at 3 and 6 month in elderly patients exclusively receiving a protein-enriched enteral diet**. ACTH, adrenocorticotropin hormone; AA, amino acids; EAA, essential amino acids. Differences between times were analyzed using the Wilcoxon test, considering P < 0.05 to be significant. In each group, superscript letters were significantly different.

As shown in Figure 1A, reductions from baseline values were observed in plasma ACTH (baseline, 15.37 ± 1.08 pmol/L; 3 months, 9.85 ± 0.83 pmol/L; 6 months, 9.56 ± 1.18 pmol/L; *p *= 0.004) and in plasma cortisol (baseline, 423 ± 23 nmol/L; 3 months, 361 ± 35 nmol/L, 6 months, (257 ± 29 nmol/L; *p *= 0.001).

Total plasma amino acids progressively increased (Figure 1B) from 1963 ± 195 μmol/L at baseline to 2809 ± 175 μmol/L at six months (*p *= 0.008); essential amino acids increased from 558 ± 64 μmol/L at baseline to 731 ± 34 μmol/L (*p *= 0.023) at three months and remained fairly constant later on. No changes were observed over the study period in plasma urea or creatinine (Figure 1C), which remained at normal concentrations, while 3-methylhistidine (Figure 1D) was unchanged at three months but lower (*p *= 0.003) at six months. Total plasma amino acids were negatively correlated with plasma cortisol (r = -0.406, P < 0.007).

Specific plasma amino acid concentrations are reported in Table 5. Between three and six months, there were increased concentrations of leucine (*p *= 0.002), valine (*p *= 0.006), cysteine (*p *= 0.002), citrulline (*p *= 0.006), glutamine (*p *= 0.008), asparagine (*p *= 0.001), phenylalanine (*p *= 0.015) and tyrosine (*p *= 0.017). In comparison to baseline values, threonine was higher at three months (*p *= 0.017) and arginine at six months (*p *= 0.004), while proline, histidine, and methionine were higher at three months (*p *= 0.048, *p *= 0.009, and *p *= 0.012, respectively) but the same as baseline concentrations at six months. Taurine increased progressively from baseline to six months (*p *= 0.001), while alanine, glycine, isoleucine, serine, glutamic and aspartic acids, lysine, ornithine, and hydroxyproline remained largely unchanged.

**Table 5:** Plasma amino acid concentrations and indexes at baseline and at 3 and 6 months in elderly patients exclusively receiving a protein-enriched enteral diet

Amino Acids (μmol/L)	0 mo	3 mo	6 mo	P*
Aliphatic amino acids				
Alanine	317 ± 33^† ^	315 ± 39	342 ± 32	.607
Glycine	143 ± 18	183 ± 20	208 ± 18	.168
Branched-chain amino acids				
Isoleucine	38 ± 4	41 ± 6	52 ± 3	.062
Leucine	68 ± 8^a **‡**^	57 ± 6^a^	96 ± 6^b^	.002
Valine	146 ± 22^ab^	130 ± 12^a^	178 ± 12^b^	.007
Hydroxyl amino acids				
Threonine	87 ± 10^a^	132 ± 20^b^	150 ± 17^b^	.001
Serine	75 ± 7	77 ± 10	83 ± 7	.449
Sulfur amino acids				
Taurine	7 ± 1^a^	11 ± 3^b^	102 ± 11^c^	<.001
Cysteine	20 ± 5^a^	25 ± 8^a^	68 ± 6^b^	<.001
Methionine	24 ± 7^a^	59 ± 8^b^	24 ± 1^a^	.002
Acidic amino acids and amides				
Glutamic Acid	47 ± 7	46 ± 8	46 ± 8	.766
Glutamine	454 ± 64^a^	500 ± 102^a^	968 ± 123^b^	.001
Aspartic Acid	7 ± 1	7 ± 1	6 ± 1	.189
Asparagine	43 ± 8^a^	21 ± 6^a^	71 ± 6^b^	.001
Aromatic amino acids				
Phenylalanine	36 ± 5^a^	37 ± 4^a^	51 ± 3^b^	.015
Tyrosine	40 ± 4^a^	38 ± 4^a^	56 ± 5^b^	.017
Basic amino acids				
Arginine	30 ± 4^a^	44 ± 6^ab^	54 ± 5^b^	.005
Citrulline	31 ± 7^a^	25 ± 5^a^	46 ± 4^b^	.006
Histidine	81 ± 14^a^	130 ± 13^b^	85 ± 5^a^	.022
Lysine	86 ± 12	114 ± 16	91 ± 5	.189
Ornithine	77 ± 14	99 ± 21	83 ± 7	.549
Imino acids				
Hydroxyproline	12 ± 2	14 ± 2	8 ± 1	.257
Proline	190 ± 25^a^	128 ± 13^b^	194 ± 21^a^	.046
Amino Acid Indexes				
NEAA/EAA	2.54 ± 0.14^a^	2.34 ± 0.52^b^	2.93 ± 0.20^a^	.008
BCAA (μmol/L)	256 ± 32^a^	228 ± 16^a^	326 ± 21^b^	.007
Alanine/Threonine	3.85 ± 0.40^a^	3.93 ± 0.41^ab^	2.56 ± 0.31^b^	.017
Glycine/Valine	1.09 ± 0.15	2.12 ± 0.62	1.23 ± 0.12	.420
Alanine/Leucine	4.85 ± 0.40^a^	5.78 ± 0.66^a^	3.59 ± 0.27^b^	.004
Alanine/BCAA	1.36 ± 0.15^ab^	1.35 ± 0.13^a^	1.05 ± 0.07^b^	.017
Phenylalanine/Tyrosine	0.92 ± 0.07	1.00 ± 0.06	0.96 ± 0.05	.449
Cysteine/Methionine	1.46 ± 0.41^a^	0.88 ± 0.40^a^	2.80 ± 0.20^b^	.001
H Index	2.25 ± 0.22	2.52 ± 0.21	1.98 ± 0.13	.071
Whitehead Index	2.66 ± 0.25^a^	2.50 ± 0.35^a^	4.15 ± 0.43^b^	<.001

NEAA, nonessential amino acids; EAA, essential amino acids; BCAA, branched-chain amino acids; H Index, relationship between the sum of serine, glycine, and alanine concentrations and the BCAA concentration; Whitehead Index, relationship between the sum of serine, glycine, glutamine, and taurine concentrations and the sum of methionine and BCAA concentrations. Tryptophan concentrations are not reported, because much of this amino acid is bound to plasma proteins, which are eliminated during the deproteinization step in the analytical procedure

* Differences among sample times were tested using Friedman test for three repeated measures, considering P < 0.05 to be significant.

^†^x ± SEM (for all values)

‡ Differences between times were analyzed using the Wilcoxon test, considering P < 0.05 to be significant. In each group, mean values within the same row with unlike superscript letters were significantly different.

Amino acid indexes of nutritional and metabolic interest are shown in Table 5. The ratio of nonessential to essential amino acids (NEAA/EAA) was lower at three months (*p *= 0.041) but similar to baseline at six months. Between three and six months of the diet, BCAA, Cys/Met ratio, and Whitehouse index increased (*p *= 0.002, *p *= 0.002, *p *= 0.001, respectively) and the Ala/Leu and Ala/BCAA ratios decreased (*p *= 0.006, *p *= 0.012, respectively). The Ala/Thr was lower at six months than at baseline (*p *= 0.001). No changes were observed during the trial (at 3 or 6 months) in other indexes (Table 5) or in insulin, HOMA-IR, adiponectin, C-RP, IL-6, or IL-8 values (Table 6). TNF-α slightly increased from three to six months (*p *= 0.041).

**Table 6:** Insulin resistance and inflammatory biomarker concentrations at baseline and at 3 and 6 month in elderly patients exclusively receiving a protein-enriched enteral diet

	0 mo	3 mo	6 mo	P*
Insulin (mU/L)	3.12 ± 0.65^† ^	6.30 ± 1.32	4.62 ± 0.99	0.066
HOMA-IR	0.65 ± 0.13	1.41 ± 0.31	0.95 ± 0.20	0.087
Adiponectin (mg/mL)	18.7 ± 1.5	19.0 ± 1.6	20.3 ± 2.3	0.926
C-reactive protein (mg/L)	1.26 ± 0.44	1.58 ± 0.58	0.91 ± 0.28	0.886
Interleukin 6 (ng/L)	20.65 ± 4.10	29.36 ± 8.45	17.69 ± 6.29	0.424
Tumor Necrosis Factor alpha (ng/L)	4.28 ± 0.44^a **‡**^	4.53 ± 0.44^a^	5.42 ± 0.46^b^	0.007
Interleukin 8 (ng/L)	6.35 ± 0.94	6.62 ± 1.88	5.38 ± 1.39	0.165

HOMA-IR, homeostasis model assessment for insulin resistance.

* Differences between times were analyzed using the Friedman test, considering P < 0.05 to be significant.

^†^x ± SEM (for all values).

Differences between times were analyzed using the Wilcoxon test, considering P < 0.05 to be significant. In each group, mean values within the same row with unlike superscript letters were significantly different.

## Discussion

The main findings of this study were that after six months on an exclusive diet of specific protein-enriched enteral formula with a relatively high content of BCAA and other EAA, elderly patients showed a decrease in plasma cortisol concentrations and an increase in total plasma amino acid concentrations, especially of the essential amino acids leucine, valine and the conditionally essential amino acids arginine and glutamine. The plasma concentration of 3-methylhistidine, a biomarker of protein breakdown, was also decreased in these patients, but no changes were observed in creatinine, IR, or inflammation biomarkers, except for a slight increase in TNF-α at 6 months. There was a trend to a decrease in the percentage of patients with albumin levels below normal range over the duration of the diet (P = 0.06, Kendall's tau b), thus, this percentage decreased to zero after six months of the exclusive diet. These results suggest that plasma albumin concentrations can be increased by an adequate amount and quality of amino acid intake.

The Aging and Body Composition [8] study reported an association between low serum albumin concentration and greater loss of skeletal muscle mass. Comparison of the fractional synthesis rate (FSR) of albumin between young and old individuals indicated that this rate was not affected by increasing age. However, the FSR was reduced in individuals with an inadequate protein intake due to a reduction in the availability of amino acid substrate [11]. Moreover, the ingestion of 15 g/d of a mixture of EAA for 3 months in older women in a between meal fashion resulted in an increased basal FSR and improvement of the lean body mass [16], although these authors did not attempt to evaluate whether the inclusion of both EAA and NEAA would yielded the same results.

Efforts have been spent to mitigate sarcopenia in the elderly using a variety of hormones without satisfactory results [17]. However, the oral ingestion of EAA stimulates muscle protein synthesis, without affecting muscle proteolysis [10,11]. Sarcopenia has been associated with an elevated cortisol concentration, and ACTH and cortisol concentrations were reduced in our patients as a result of the protein-enriched enteral diet. Cortisol is known to stimulate the degradation and inhibit the synthesis of muscle proteins [18]. One study of 26 middle-aged and 21 elderly men found a negative relationship between cortisol and muscle strength of the knee extensor [19], and another study found a negative relationship between cortisol and high physical performance in elderly subjects.

A major increase in leucine was achieved with the present diet, suggesting a possible increase in protein synthesis. In fact, leucine and insulin are the main regulators of body protein synthesis, which is mediated by the mTOR signaling pathway, involving type 1 phosphoinositide-3 kinase (PI3K) and protein kinase B (PKB/Akt) [20]. Studies in old rats showed that protein synthesis was stimulated by supplementation with leucine [21]. The same effect was observed by Rieu *et al *[22] in the elderly and by Koopman *et al. *in both elderly and young individuals [10]. Recent studies indicated that a novel class type 3 PI3K, vacuolar protein sorting 34 (Vps34), which is stimulated by amino acids, primarily by leucine, increases the production of PI3P. PI3P recruits proteins containing FYVE or PX to enhance phosphorylation and activation of mTOR, which finally inhibits 4EBP-1 and activates S6K1 [20]. Compared with the composition of the FAO/WHO reference protein intake [23], the enteral diet used in the present study supplied about twice the content of BCAA.

Some EAA and NEAA, including arginine, glutamine, and proline, are important regulators of key metabolic pathways that are necessary for maintenance, growth, reproduction, and immunity in organisms, enhancing protein secretion and improving health [24]. Glutamine and proline metabolisms are interconnected *via *glutamate and pyrroline-5-carboxilate and both amino acids can serve as an important precursor for arginine [25]. Glutamine participates in protein synthesis, gluconeogenesis, inter-organ nitrogen transfer, nucleic acid biosynthesis, immune response, regulation of cellular redox state, and ammonia detoxification [26] by shuttling ammonia to the gut and kidney for excretion and as a precursor to arginine and urea synthesis [25]. Arginine is synthesized in the liver, but there is no net synthesis *via *the hepatic urea cycle, because there is high arginase activity to hydrolyze this amino acid. In adults, the endogenous synthesis of arginine involves the intestinal-renal axis [27]. Citrulline is synthesized from glutamine, glutamic acid, and proline in the enterocyte, released from the small intestine, and taken up primarily by kidneys for arginine production. Besides the kidney, citrulline is readily converted into arginine in nearly all types of cells [27]. In our study, citrulline increased from 3 to 6 month of the enteral diet, indicating that it provided sufficient substrates (i.e., glutamate and glutamine) for its synthesis and explaining the increased plasma arginine levels. The protein source used in the present study is not only rich in glutamine but also in proline (high proportion of casein) and arginine (pea protein content).

Other amino acids, such as those containing sulfur, are of importance in health. The major end-products of methionine and cysteine metabolism are glutathione (GSH), homocysteine, and taurine, which have key roles in the antioxidant defense system and in the intestinal immune response. GSH is the major intracellular low-molecular weight thiol and plays an important part in regulating the homeostasis of free radicals and cytoprotective events [28]. The increase in cysteine levels between three to six months on our diet is of interest, because cysteine is the rate-limiting amino acid for GSH synthesis. There was also an increase in taurine, which participates in maintaining the antioxidant system and is the main component of the free amino acid pool of lymphocytes, indicating its potential importance in immune and proinflamatory responses. Taurine is also involved in detoxification, membrane stabilization, and retinal and cardiac function [26]. Hence, dietary supplementation using a protein source with a high biological value and an adequate distribution of both essential and semi-essential amino acids [13] appears to be a good strategy to maintain plasma amino acid levels and reduce glucocorticoids and muscle breakdown in the elderly. Thus, a decrease in plasma 3-methylhistidine intake was achieved with our enteral diet, indicating a lesser protein breakdown, since this metabolite cannot be reincorporated into proteins after its release by protein degradation. Furthermore, there was no change in creatinine, a biomarker of protein degradation, suggesting maintenance of the muscular mass without reduction of the arginine pool.

An inflammatory state is usually present in sarcopenia, although it is more frequent in cancer patients. In the elderly, however, the degree to which sarcopenia is associated with a reduced insulin response and/or the presence of inflammatory factors is not clear. The increase in IL-6 with age is well documented. Bautman *et al. *observed an association between lower muscle performance and higher IL-6 levels in hospitalized geriatric patients [29], and Yende *et al *reported low quadriceps strength in elderly individuals with high levels of IL-6 and TNF-α [30]. In the present study, the enteral diet produced no changes in IR or increase in inflammation biomarkers, (IL-6, IL-8 and C-RP), except in the case of TNF-α which increased but remaining within normal ranges, indicating that it did not induce an inflammatory state or influence levels of pro-inflammatory cytokines, while maintaining the homeostasis of nitrogen metabolism.

Our study has some limitations, including the large number of subjects who did not complete it, although its duration was sufficient for some specific metabolic changes to be observed. We were not able to measure the FSR or other parameters directly related to protein synthesis and muscle strength because of the clinical condition of our patients, with the majority having cognitive deficits.

## Conclusion

Based on our findings, we can conclude that an enteral diet with a specific blend of protein source with a high proportion of BCCA, glutamate/glutamine, and arginine, among other essential and conditionally essential amino acids, increases plasma essential amino acids, especially leucine, and decreases cortisol and 3-methylhistidine, suggesting that protein synthesis is enhanced and protein degradation is reduced.

## Abbreviations

IL: interleukin; TNF-α: tumor necrosis factor alpha; IR: insulin resistance; EAA: essential amino acids; EN: enteral nutrition; BCAA: branched-chain amino acids; PER protein efficiency ratio; MAC: mid-arm circumference; TS: tricipital skinfold; CV: coefficient of variation; HOMA: homeostatic assessment model; C-RP: C-reactive protein; PETIA: particle-enhanced turbidimetric immunoassay; HPLC: high-performance liquid chromatography; ACTH: adrenocorticotropin hormone; SEM standard error of the mean; NEAA: nonessential amino acids; FSR: fractional synthesis rate; PI3K: phosphoinositide-3 kinase; PKB/Akt: protein kinase B; Vps34: vacuolar protein sorting 34; GSH: glutathione.

## Competing interests

MP is a member of the Research and Development Department of Vegenat, the company that funded the present study, and she was involved in the development of the product T-Diet Plus. AG has no contractual relationship with Vegenat but participated in the product design as part of a research contract between the University of Granada Foundation and Vegenat (Contract n° 2388).

## Authors' contributions

JO carried out the study, the statistical analyses and participated in the drafting of the manuscript; MDM was involved in the data analyses; RP participated in the amino acid and hormone analyses; CMA took part in the inflammatory biomarker analyses; MP conceived of the study and was responsible for the design of the product; RMT was involved in the study design and coordination of institutions; APC participated in the design of the product and the study; AG participated in the design of the product, was responsible for coordinating the study, and collaborated in the data analysis and drafting of the manuscript. All authors read and approved the final manuscript.

## References

[B1] Lee CE McArdle A Griffiths RD The role of hormones, cytokines and heat shock proteins during age-related muscle loss Clin Nutr 2007 26 524 534 10.1016/j.clnu.2007.05.005 17590243

[B2] Combaret L Dardevet D Béchet D Taillandier D Mosoni L Attaix D Skeletal muscle proteolysis in aging Curr Opin Clin Nutr Metab Care 2009 12 37 41 10.1097/MCO.0b013e32831b9c31 19057185

[B3] Griffiths RD Muscle, survival and elderly ICU patients Nutr 1996 12 456 458 Dr RD Griffiths BSC, MD, FRCP 10.1016/S0899-9007(96)00141-4 8875547

[B4] Timmerman KL Volpi E Amino acid metabolism and regulatory effects in aging Curr Opin Clin Nutr Metab Care 2008 11 45 49 10.1097/MCO.0b013e3282f2a592 18090658 PMC2804959

[B5] Thomas DR Loss of skeletal muscle mass in aging: Examining the relationship of starvation, sarcopenia and cachexia Clin Nutr 2007 26 389 399 10.1016/j.clnu.2007.03.008 17499396

[B6] Paddon-Jones D Sheffield-Moore M Cree MG Hewlings SJ Aarsland A Wolfe RR Ferrando AA Atrophy and impaired muscle protein synthesis during prolonged inactivity and stress J Clin Endocrinol Metab 2006 91 4836 4841 10.1210/jc.2006-0651 16984982

[B7] Hamrahiam AH Seni TS Arafah BM Measurement of serum free cortisol in critically ill patients N Engl J Med 2004 350 1629 1638 10.1056/NEJMoa020266 15084695

[B8] Visser M Kritchevsky SB Newman AB Goodpaster BH Tylavsky FA Nevitt MC Harris TB Lower serum albumin concentration and change in muscle mass: the Health and Body Composition study Am J Clin Nutr 2005 82 531 537 16155264 10.1093/ajcn.82.3.531

[B9] Jarosz PA Bellar A Sarcopenic obesity: an emerging cause of frailty in older adults Geriatr Nurs 2009 30 64 70 10.1016/j.gerinurse.2008.02.010 19226689

[B10] Koopman R Verdijk L Manders RJ Gijsen AP Gorselink M Pijpers E Wagenmakers AJ van Loon LJ Co-ingestion of protein and leucine stimulates muscle protein synthesis rates to the same extent in young and elderly lean men Am J Clin Nutr 2006 84 623 632 16960178 10.1093/ajcn/84.3.623

[B11] Thalacker-Mercer AE Johnson CA Yarasheski KE Carnell NS Campbell WW Nutrient ingestion, protein intake, and sex but not age, affect the albumin synthesis rate in humans J Nutr 2007 137 1734 1740 17585023 10.1093/jn/137.7.1734 PMC3885871

[B12] Paddon-Jones D Sheffield-Moore M Katsanos CS Zhang XJ Wolfe RR Differential stimulation of muscle protein synthesis in elderly humans following isocaloric ingestion of amino acids or whey protein Exp Gerontol 2006 41 215 219 10.1016/j.exger.2005.10.006 16310330

[B13] Olza J Porres J Martínez de Victoria E Gil A Evaluación biológica de la calidad de una mezcla de proteínas para uso en nutrición enteral Nutri Hosp 2008 3 206 211 18560696

[B14] Esquius M Schwartz S López Hellín J Andreu AL García E Anthropometric reference parameters for the aged population Med Clin (Barc) 1993 100 692 698 8492597

[B15] Le Boucher J Charret C Coudray-Lucas C Giboudeau J Cynober L Amino acid determination in biological fluids by automated ion-exchange chromatography: performance of Hitachi L-8500A Clin Chem 1997 43 1421 1428 9267323

[B16] Dillon EL Sheffield-Moore M Paddon-Jones D Gilkison C Sanford AP Casperson SL Jiang J Chinkes DL Urban RJ Amino acid supplementation increases lean body mass, basal muscle protein synthesis, and insulin-like growth factor-I expression in older women J Clin Endocrinol Metab 2009 94 1630 1637 10.1210/jc.2008-1564 19208731 PMC2684480

[B17] Nair KS Rizza RA O'Brien P Dhatariya K Short KR Nehra A Vittone JL Klee GG Basu A Basu R Cobelli C Toffolo G Dalla Man C Tindall DJ Melton LJ Smith GE Khosla S Jensen MD DHEA in elderly women and DHEA or testosterone in elderly men N Engl J Med 2006 355 1647 1659 10.1056/NEJMoa054629 17050889

[B18] Peeters GMEE van Schoor NM van Rossumt EFC Visser M Lips P The relationship between cortisol, muscle mass and muscle strength in older persons and the role of genetic variations in the glucocorticoid receptor Clin Endcrinol 2008 69 673 682 10.1111/j.1365-2265.2008.03212.x 18248637

[B19] Izquierdo M Häkkinen K Antón A Garrues M Ibañez J Ruesta M Gorostiaga EM Maximal strength and power, endurance performance, and serum hormones in middle-aged and elderly men Med Sci Sports Exerc 2001 33 1577 1587 10.1097/00005768-200109000-00022 11528348

[B20] Fujita S Dreyer HC Drummond MJ Glynn EL Cadenas JG Yoshizawa F Volpi E Rasmussen BB Nutrient signaling in the regulation of human muscle protein synthesis J Physiol 2007 582 813 823 10.1113/jphysiol.2007.134593 17478528 PMC2075348

[B21] Rieu I Sornet C Bayle G Prugnaud J Pouyet C Balage M Papet I Grizard J Dardevet D Leucine-supplemented meal feeding for ten days beneficially affects postprandial muscle protein synthesis in old rats J Nutr 2003 133 1198 1205 12672943 10.1093/jn/133.4.1198

[B22] Rieu I Balague M Sornet C Giraudet C Pujos E Grizard J Mosoni L Dardevet D Leucine supplementation improves muscle protein synthesis in elderly men independently of hyperaminoacidaemia J Physiol 2006 575 305 315 10.1113/jphysiol.2006.110742 16777941 PMC1819434

[B23] FAO/WHO Protein quality evaluation: report of the Joint FAO/WHO Expert Consultation FAO Food and Nutrition Paper 51 1989 Bethesda: FAO

[B24] Wu G Amino acids: metabolism, functions, and nutrition Amino Acids 2009 37 1 17 10.1007/s00726-009-0269-0 19301095

[B25] Bertolo RF Burrin DG Comparative aspects of tissue glutamine and proline metabolism J Nutr 2008 138 2032S 9S 18806120 10.1093/jn/138.10.2032S

[B26] Wang WW Qiao SY Li DF Amino acids and gut function Amino Acids 2009 37 105 110 10.1007/s00726-008-0152-4 18670730

[B27] Wu G Bazer FW Davis TA Kim SW Li P Marc Rhoads J Carey Satterfield M Smith SB Spencer TE Yin Y Arginine metabolism and nutrition in growth, health and disease Amino Acids 2009 37 153 168 10.1007/s00726-008-0210-y 19030957 PMC2677116

[B28] Grimble RF The effect of sulphur amino acids intake on immune function in humans J Nutr 2006 136 Supp 6 1660 1665 10.1093/jn/136.6.1660S 16702336

[B29] Bautmans I Njemini R Lambert M Demanet C Mets T Circulating acute phase mediators and skeletal muscle performance in hospitalized geriatric patients J Gerontol A Biol Sci Med Sci 2005 60 361 367 15860475 10.1093/gerona/60.3.361

[B30] Yende S Waterer GW Tolley EA Newman AB Bauer DC Taaffe DR Jensen R Crapo R Rubin S Nevitt M Simonsick EM Satterfield S Harris T Kritchevsky SB Inflammatory markers are associated with ventilatory limitation and muscle dysfunction in obstructive lung disease in well functioning elderly subjects Thorax 2006 61 1 3 10.1136/thx.2006.065144 16284220 PMC2080698

